# Duration of Cellular and Humoral Responses after Quadrivalent Human Papillomavirus Vaccination in Healthy Female Adults with or without Prior Type 16 and/or 18 Exposure

**DOI:** 10.3390/vaccines8030348

**Published:** 2020-06-30

**Authors:** Lilin Lai, Kevin Ault, Nadine Rouphael, Allison Beck, Briyana Domjahn, Yongxian Xu, Evan J. Anderson, Andrew Cheng, Aya Nakamura, Rebecca J. Hoagland, Colleen Kelley, Srilatha Edupuganti, Karen Mask, Mirjana Nesin, Elizabeth R. Unger, Gitika Panicker, Hagit David, Mark J. Mulligan

**Affiliations:** 1 The Hope Clinic of the Emory Vaccine Center, Division of Infectious Diseases, Department of Medicine, Emory University School of Medicine, 500 Irvin Court Suite 200, Decatur, GA 30030, USA; Lilin.Lai@nyulangone.org (L.L.); Allison.Beck@NIH.gov (A.B.); Briyana.domjahn@gmail.com (B.D.); y.xu@emory.edu (Y.X.); ahchen3@emory.edu (A.C.); colleen.kelley@emory.edu (C.K.); sedupug@emory.edu (S.E.); Karen.Mask@gmail.com (K.M.); Mark.mulligan@nyulangone.org (M.J.M.); 2 Department of Obstetrics and Gynecology, University of Kansas Medical Center, 3901 Rainbow Blvd, Kansas City, KS 66160, USA; kault2@kumc.edu; 3 Department of Pediatrics, Emory University School of Medicine, 2015 Uppergate Drive NE, Atlanta, GA 30322, USA; evanderson@emory.edu; 4 The EMMES Company, LLC, 401 N. Washington St., Suite 700, Rockville, MD 20850, USA; anakamura@emmes.com; 5 Cota Enterprises, 16570 46th Street, McLouth, KS 66054, USA; r.hoagland@cotaenterprises.com; 6 Division of Microbiology and Infectious Diseases, NIAID, NIH, 5601 Fishers Lane, Rockville, MD 20892-9825, USA; Nesinm@me.com (M.N.); hagit.s.david@gmail.com (H.D.); 7 Division of High-Consequence Pathogens and Pathology, Centers for Disease Control and Prevention, 1600 Clifton Rd NE, Atlanta, GA 30329, USA; eru0@cdc.gov (E.R.U.); dhv1@cdc.gov (G.P.); 8 New York University Langone Vaccine Center, Alexandria Center for Life Sciences (West Tower), 430 E 29th St, Room 304, New York, NY 10016, USA

**Keywords:** HPV vaccine, memory B cell responses, T cell responses, ELISA, pseudovirion neutralization assay

## Abstract

Human papillomavirus virus (HPV) vaccines aim to provide durable protection and are ideal to study the association of cellular with humoral responses. We assessed the duration and characteristics of immune responses provided by the quadrivalent HPV (4vHPV) vaccine in healthy female adults with or without prior exposure with type 16 and 18 HPV. In a prospective cohort, vaccine naïve females received three doses of 4vHPV vaccine and were followed for two years to assess cellular (intracellular cytokine staining, proliferation and B cell ELISpot assays) and humoral (multiplex L1/L2 viral-like particles (VLP) and M4 ELISAs) responses. Frequencies of vaccine-specific CD4+ T cells correlated with antibody responses. Higher HPV antibody titers were found at all time points in participants previously exposed to HPV, except for anti-HPV-18 at Day 187 (one week post the third vaccination). Retrospective cohorts enrolled females who had previously received two or three 4vHPV doses and tested antibody titers by M4 ELISA and pseudovirion neutralization assay along with memory B cells (MBCs). Almost all women enrolled in a retrospective cohort with two prior doses and all women enrolled in a retrospective cohort with three prior doses had sustained antibody and memory responses. Our findings indicate that HPV vaccination induces a long-lasting, robust cellular and humoral immune responses.

## 1. Introduction

Human papillomaviruses (HPV) types 16 and 18 are the most important high-risk HPV types, causing approximately 70% of cervical cancers worldwide. The first two HPV vaccines developed, the bivalent AS04-adjuvanted HPV-16/18 vaccine (2vHPV, Cervarix, GlaxoSmithKline PLC) and the quadrivalent alum-adjuvanted HPV 6/11/16/18 vaccine (4vHPV, Gardasil, Merck & Co., Inc.) were licensed in 2006 for the prevention of cervical cancer and high-grade precursor lesions. In 2015, Gardasil 9 (9vHPV) targeting HPV 6, 11, 16, 18, 31, 33, 45, 52 and 58 was licensed; this is currently the only vaccine available in the US. When administered as three-dose schedules, these vaccines demonstrated high vaccine efficacy (>96%) against cervical cancer precursors in HPV-naïve individuals and induced high levels of type-specific antibodies that persist for over nine years [1,2,3,4,5,6]. The high immune responses and effectiveness of the HPV vaccines led the Centers for Disease Control and Prevention (CDC, Atlanta, GA, USA) to revise their recommendations in 2016 from administering three doses of HPV vaccine over six months to two doses given 6–12 months apart for individuals younger than 15 years of age [7,8] after studies found non-inferior antibody (Ab) responses with reduced-dose HPV schedules. HPV vaccines have been found to induce herd immunity after the introduction of national programs, with significant declines in the prevalence of vaccine-type virus both in vaccinated and unvaccinated women [9,10].

However, important questions have not been fully answered by earlier clinical trials, and immune correlates of protection have not been established. Vaccines that induce long-term protection are usually characterized by the generation of immune memory. Previous studies showed circulating HPV-16 and -18 specific memory B cells can be detected after vaccination with either 4vHPV or 2vHPV vaccines [4,11], but the duration of follow-up was 30 and seven months, respectively. In addition, the role of T cell immunity in the prevention and control of HPV infection is not well established. Immune responses after vaccination in previously HPV-exposed versus HPV-naïve individuals have also not been directly compared [12]. 

In this study, we report results from a prospective analysis of T and B cell responses following 4vHPV vaccination in HPV-naïve and HPV-exposed women and analyses of immune memory responses in two previously vaccinated cohorts (labeled as retrospective cohorts). Our overall goal was to provide further insights on the quality, magnitude, and duration of cellular and humoral responses induced by the HPV vaccine.

## 2. Materials and Methods

### 2.1. Study Design and Vaccine

Subjects for all 3 cohorts were enrolled from April 2012 through May 2015 at the Hope Clinic of Emory Vaccine Center and Kaiser Permanente in Georgia, USA. In the prospective cohort, a total of 47 healthy women aged 18–26 years were enrolled and vaccinated with three doses of 4vHPV intramuscularly at months 0, 1 and 6. Participants were followed for two years. Results of prevaccination vaginal swabs for HPV DNA detection and serum ELISA antibody titers were used to categorize subjects’ prior exposure to HPV-16 or HPV-18 (assay methods detailed below). Blood for immunological testing was collected at baseline (Day 0), Day 67, Day 187, Day 365, Day 545 and Day 730 postenrollment. For the two retrospective cohorts, healthy females aged 18 to 30 years were enrolled, 78 who had previously received two 4vHPV vaccinations (Retro 2 cohort), and another 78 subjects who had previously received three 4vHPV vaccinations (Retro 3 cohort). Medical records were requested to confirm the number of doses of HPV and date of administration. Subjects from both retrospective cohorts attended only a single study visit during which blood samples were collected for antibody and memory B cell ELISpot assays. Subjects in the Retro 2 cohort also received a third dose of 4vHPV vaccine intramuscularly at enrollment as three doses were recommended for all vaccines at the time of the study. This study was registered at ClinicalTrials.gov under identifier NCT01505049. The study was approved by the Emory University Institutional Review Board (IRB00046117 approved effective November 02, 2011) and written informed consent was provided by subjects prior to study participation

### 2.2. Cellular Immunological Assays

Peripheral blood mononuclear cells (PBMC) were separated from heparinized whole blood using a Ficoll-Hypaque gradient and stored in liquid nitrogen. Cryopreserved PBMCs were later thawed in a 37 °C water bath and washed. Before use, cells were counted and checked for viability by Trypan blue dye exclusion.

Peptide pools for intracellular cytokine staining (ICS) and Carboxyfluorescein Succinimidyl Ester (CFSE) T cell proliferation assays were synthesized by JPT Peptide Technologies (Berlin, Germany). These consisted of 15-mers with 11 amino acid overlaps spanning the L1 region of HPV16 HPV18 protein region of HPV-16 and-18 (GenBank: #AAC09292 and #AAQ92369, respectively). The HPV-specific response was determined by intracellular cytokine (IFNγ, IL-2, IL-4 and/or IL-21) expression or CFSE dilution in CD4+ T cells upon ex vivo peptide stimulation. To define the percentage of vaccine-induced CD4+ T cell responses, Day 0 antigen (Ag)-specific responses from subjects who were shown to be seronegative and DNA-negative for HPV-16 and HPV-18 at baseline were used to establish a positive cut-off value corresponding to the 95th percentile of the Ag-specific responses in this HPV-negative population (HPV-naïve subjects). The positive responder was defined as subjects whose value was greater than the baseline value and with >180 HPV-16+ or >296 HPV-18+ CD4 T cells per million of total CD4 T cells.

For analyses involving memory B cells and cytokine response, a threshold value was calculated for each antigen (HPV-16 and HPV-18). The threshold value was calculated by taking the mean plus 3 standard deviations of the response value at Day 0 (baseline) for all subjects who were ELISA HPV seronegative and vaginal swab DNA-negative at baseline.

HPV16 and 18 L1/L2 viral-like particles (VLPs) were provided by the CDC for B cell enzyme-linked immunospot (ELISpot) assay. MBC response was reported as the number of HPV-16- or HPV-18-specific IgG-secreting B cells per million total IgG-secreting B cells. The associated 95% CI for the number of responders was calculated using a two-sided exact Clopper–Pearson method. A subject was classified as a responder in the prospective cohort if she had a postbaseline value greater than the baseline value and greater than the threshold value (negative cut-off) for the assay.

Additional experimental methods for ICS, CFSE and MBC ELISpot assays are provided in Appendix A.

### 2.3. Multiplexed Enzyme-Linked Immunosorbent Assay (M4ELISA)

Antibody responses to HPV-16 and -18 were measured using the multiplex M4ELISA as previously reported [13,14]. Further experimental methods are provided in the Appendix A.

### 2.4. Pseudovirion-Based Neutralization Assay Using Secreted Alkaline Phosphatase (PBNA)

HPV-16 and -18 pseudovirions were produced as previously described [15]. The assay was performed as described, with a few modifications [16]. Serum samples were serially diluted 2-fold in neutralization buffer, starting at 1:50. Assay controls included a pooled serum sample positive for HPV 16 and 18 antibodies, heparin (1 mg/mL; Sigma-Aldrich, St. Louis, MO, USA) and neutralization buffer alone. Secreted alkaline phosphatase (SEAP) activity in the samples was measured using the Great EscAPE SEAP kits (ClonTech, Mountainview, CA, USA) as per the manufacturer’s instructions. Titers were calculated as the reciprocal of the highest dilution of the sample that resulted in a 50% reduction of SEAP activity compared to that of the HPV 16/18 pseudovirus in neutralization buffer alone. A titer of 50 or higher was considered seropositive, as described in the WHO HPV Labnet Manual [17].

### 2.5. HPV Detection in Cervical Swab Specimens

Self-collected vaginal swabs were placed in STM vials (Qiagen, Hilden, Germany), extracted with MagNa Pure (Roche, Basel, Switzerland) and tested with RUO Linear Array HPV typing assay following previously reported methods [18].

### 2.6. Statistical Analysis

The absolute memory response and the percentage of subjects who were responders were summarized for HPV-16 and HPV-18. A subject was defined as a responder if her memory response was greater than the threshold value (negative cut-off). The minimum, maximum geometric mean frequency, geometric standard deviation and a 95% confidence interval were summarized for HPV-naïve and HPV-exposed groups for HPV-16 and HPV-18 in each cohort.

A comparison of the absolute response was made using a two-sided, two-sample t-test. The difference in the absolute mean response was calculated on the log 10 scale and back-transformed for reporting. Both the geometric mean difference and two-sided 95% CI were reported. A comparison of the percentage of subjects who were responders was made using a two-sided Fisher’s Exact test. The difference in proportions and two-sided 95% CI for the difference was reported.

Vaccine-induced CD4+ T cell responses to HPV-16 and -18 were assessed as total cytokine-expressing cells (producing IFNγ, IL-2, IL-4 and/or IL-21) at 7 days post-second and third vaccinations and compared with baseline levels. The magnitude of the correlation between CD4+ T cell responses and antibody titers at study time points was determined by a Spearman Rank Correlation Test.

Additional statistical information is provided in Appendix A.

## 3. Results

### 3.1. Study Populations

**Figure 1:**
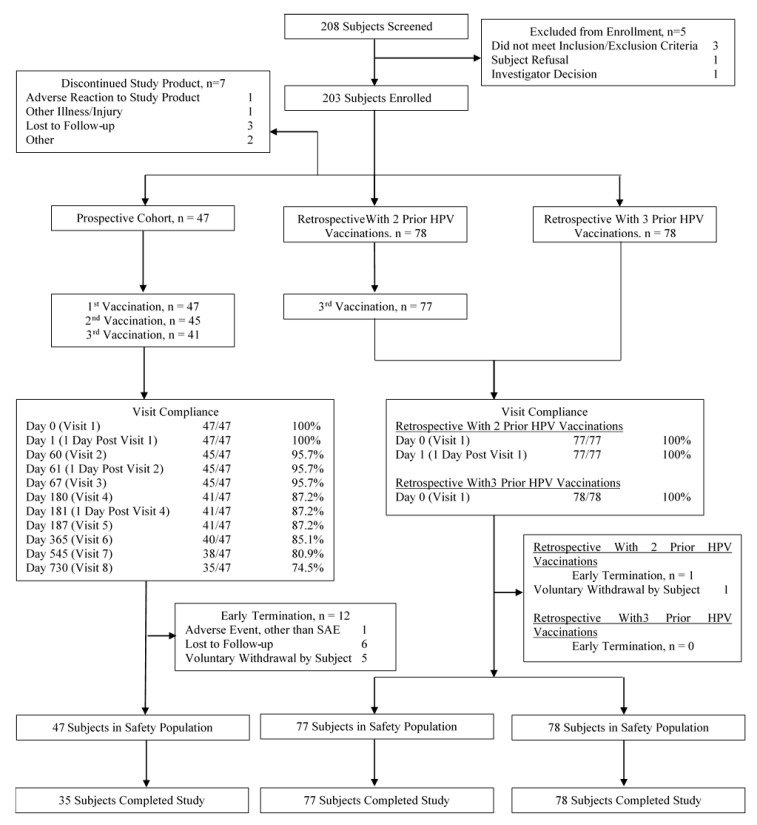
Disposition of study subjects-consort flow diagram. n: the number of subjects.

We enrolled a total of 203 female subjects for the three studies, specifically 47 in the prospective cohort, 78 in the Retro 2 cohort, and 78 in the Retro 3 cohort (Table 1 and Figure 1). The median age was 25 years and was similar across the cohorts. Most subjects were non-Hispanic (96.0%) and a majority were white (65.0%). The Retro 3 cohort (received 3 prior HPV vaccinations) had a higher proportion of whites (82%) compared to the prospective cohort (51%) and the Retro 2 cohort (56%). The elapsed time from the last vaccinations differed between the Retro 2 vs. Retro 3 cohorts with the medians being 475 (16 months) days vs. 1608 (54 months) days, respectively. In the prospective cohort, baseline DNA and ELISA antibody assays were negative (defined as HPV-naïve) in 36 of 47 (77%) and 37 of 47 (79%) participants for HPV-16 and HPV-18, respectively (Table 1).

### 3.2. Frequencies of HPV-Specific CD4+ T Cells

**Figure 2:**
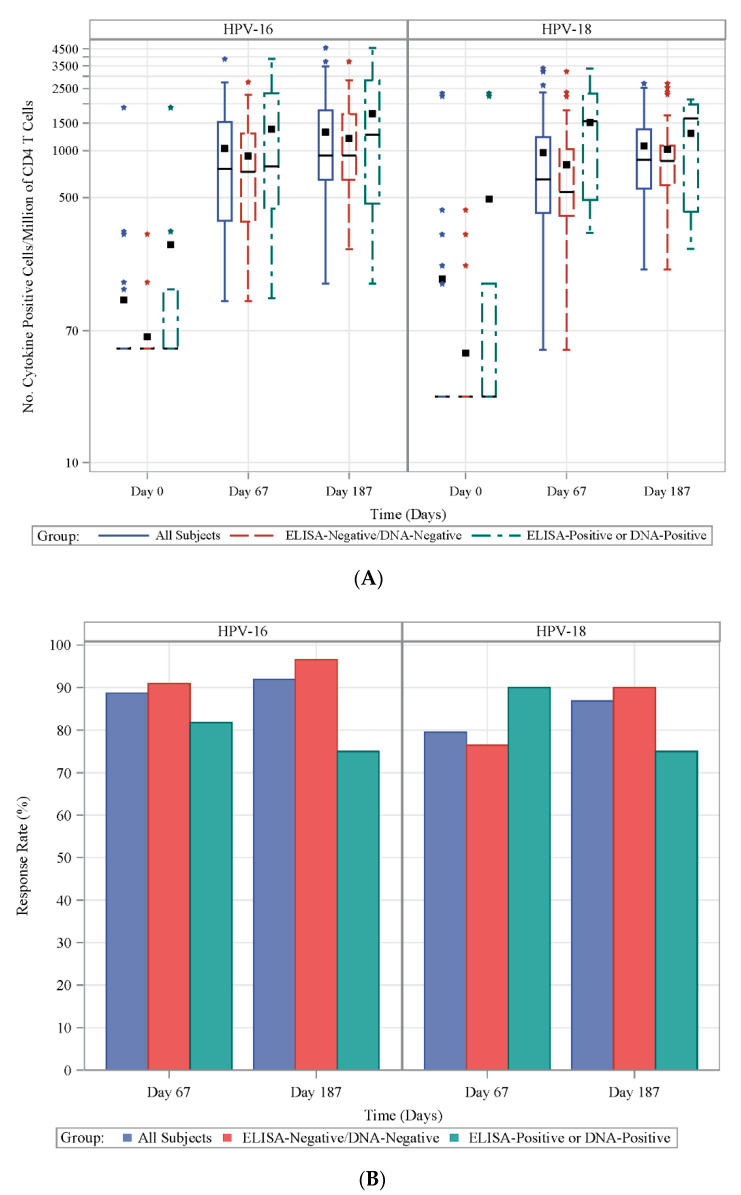
Frequency and proportion of responders of HPV-specific CD4+ T cell induced by 4vHPV vaccination. (**A**) Frequency of HPV-16- (left panel) and HPV-18 (right panel)-specific CD4+ T cell response at Days 0, 67 and 187 in the prospective cohort for all (solid blue line), HPV-naïve (dashed red line) and HPV-exposed (dashed green line) subjects expressed as the number of total IFNγ, IL-2, IL-4 and IL-21 expressing cells per millions of total CD4 T cells; (**B**) proportion of responders for HPV-16- (left panel) and HPV-18 (right panel)-specific CD4+ T cell response at Days 67 and 187 in prospective cohort defined as subjects whose value was greater than the baseline value and with >180 HPV-16+ or >296 HPV-18+ CD4 T cells per million of total CD4 T cells for all (blue bar), HPV-naïve (red bar) and HPV-exposed (green bar) subjects.

Among the HPV-naïve subjects in the prospective cohort, the postvaccination CD4+ T cell response magnitudes and proportions of positive responders were comparable. Overall, frequencies of HPV-specific CD4+ T cells significantly increased (relative to prevaccination) at seven days post-second vaccination (Day 67) with geometric means (GM) of 718 vs. 66 (*p* = 0.0003) and 683 vs. 40 (*p* < 0.0001) for total cytokine-producing CD4+ T cells per 10^6^ CD4+ T cells against HPV-16 and HPV-18, respectively. At Day 7 post-third vaccination (Day 187), magnitudes of HPV-specific CD4+ T cells increased relative to Day 67 for both HPV-16 (983), and HPV-18 (867), but were not statistically significant (Figure 2A; Table S1).

Subjects who were HPV-exposed had higher frequencies of cytokine-producing CD4+ T cells at baseline as expected (GM number of IL-2, IL-4, IL-21, and/or INF-γ positive cells per million CD4+ T cells were 59 vs. 94 against HPV-16 and 33 vs. 76 against HPV-18) when compared to naïve subjects. After vaccination, more CD4+ T cells expressed cytokines against HPV-16 and HPV-18 at each time point in HPV-exposed subjects compared with HPV-naïve subjects, but differences were only statistically significant for HPV-18 at Day 67 (*p* = 0.039; Figure 2A; Table S1).

Approximately 89% and 92% of subjects responded to HPV-16 at Day 67 (Day 7 post-second vaccination) and Day 187 (Day 7 post-third vaccination), respectively, and approximately 80% and 87% of subjects responded to HPV-18 at Day 67 and Day 187, respectively (Figure 2B).

### 3.3. Antibody Response to Vaccination in Prospective Cohort

**Figure 3:**
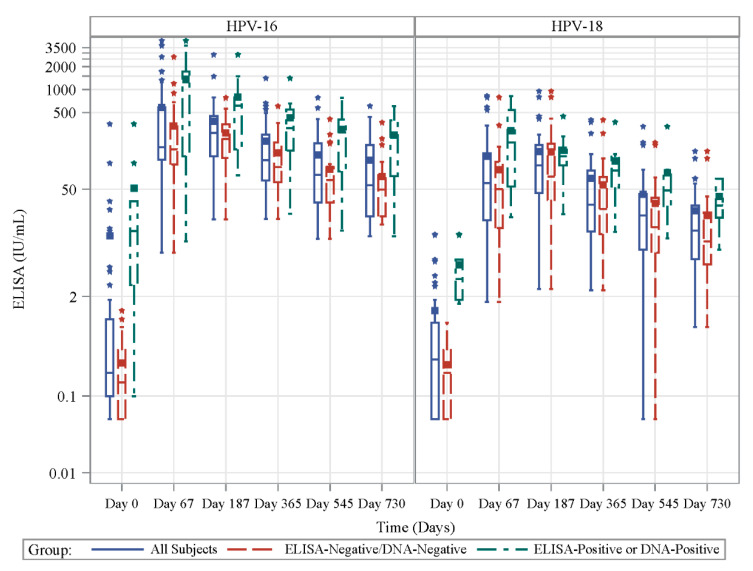
Antibody levels measured by ELISA increased in response to HPV vaccination. HPV-16- (left side) specific and HPV-18-(right side) specific antibody (Ab) titers detected by ELISA (IU/mL) at Days 0, 67, 187, 365, 545 and 730 in prospective cohort for all (solid blue line), HPV-naïve (dashed red line) and HPV-exposed(dashed green line) subjects.

Antibody levels as measured by ELISA increased from baseline for all subjects and 100% of subjects were seropositive for HPV 16 and 18 following vaccination. Regardless of HPV type, geometric mean increased through Day 187, and remained measurable through Day 730 for both HPV types. (Figure 3; Table S2).

As expected, baseline antibody levels were higher in the HPV-exposed group than in the HPV-naïve group (HPV-16 geometric means of 9.8 and 0.16 IU/mL, respectively, *p* < 0.001; (HPV-18 geometric means of 3.9 and 0.16 IU/mL, respectively, *p* < 0.0001; Figure 3; Table S2, Table 2). This significant difference persisted at all postvaccination time points measured for both HPV types except for HPV-18 at Day 187. Interestingly, although we found the virus-specific IgG levels increased from baseline to Day 67 (after the second dose) for both HPV-naïve and HPV-exposed groups (Naïve: geometric mean increased from 0.16 to 165 for HPV-16 and 0.16 to 39 IU/mL for HPV-18. Exposed: geometric means 9.8 to 499 for HPV-16 and 3.9 to 165 IU/mL for HPV-18), only the HPV-naïve group showed further increases by Day 187 (after the third dose) 165 to 208 IU/mL for HPV-16 and 39 to 74 IU/mL for HPV-18l IU/mL).

### 3.4. T Lymphocyte Proliferative Capacity

In contrast to the ICS and MBC ELISpot assays, where exposed individuals had detectable responses prevaccination, no HPV type-specific CD4+ T cell proliferation was detected at enrollment (prevaccination) in either the HPV-naïve or exposed groups using the CFSE dilution assay (Table S3). The CD4+ T cell proliferation assay GM trended non-significantly higher for HPV-16 than HPV-18 across all postvaccination study days assayed (GMof % CFSE low cells among all CD4+ T cells for HPV-16 vs. -18: 0.68 vs. 0.56, 0.95 vs. 0.69, and 1.14 vs. 0.93% at Days 365, 545 and 730, respectively). Quite remarkably, the proliferative potential of the HPV type-specific CD4+ T cells (for both HPV-16 and -18) continued to increase at Day 730, two years after the first vaccination (Table S3).

### 3.5. Correlations between CD4+ T Cell and Ab Responses

For HPV-16, the proportion of cytokine-producing HPV-16-specific CD4+ T cells at Days 67 and 187 significantly correlated with ELISA Ab titers at all time points (Table 3). The correlations were less consistent for HPV-18-specific total cytokine-secreting CD4+ T cells; the proportion at Day 67 correlated with Ab levels at time points other than Day 730, while the Day 187 HPV-18-specific CD4+ T response significantly correlated only with the Ab response on the same day. When analyzed by a subset of prior HPV exposure, some CD4+ T cell and antibody correlations were no longer significant (data not shown).

### 3.6. Antibody Response to Vaccination-Retrospective Cohort

**Figure 4:**
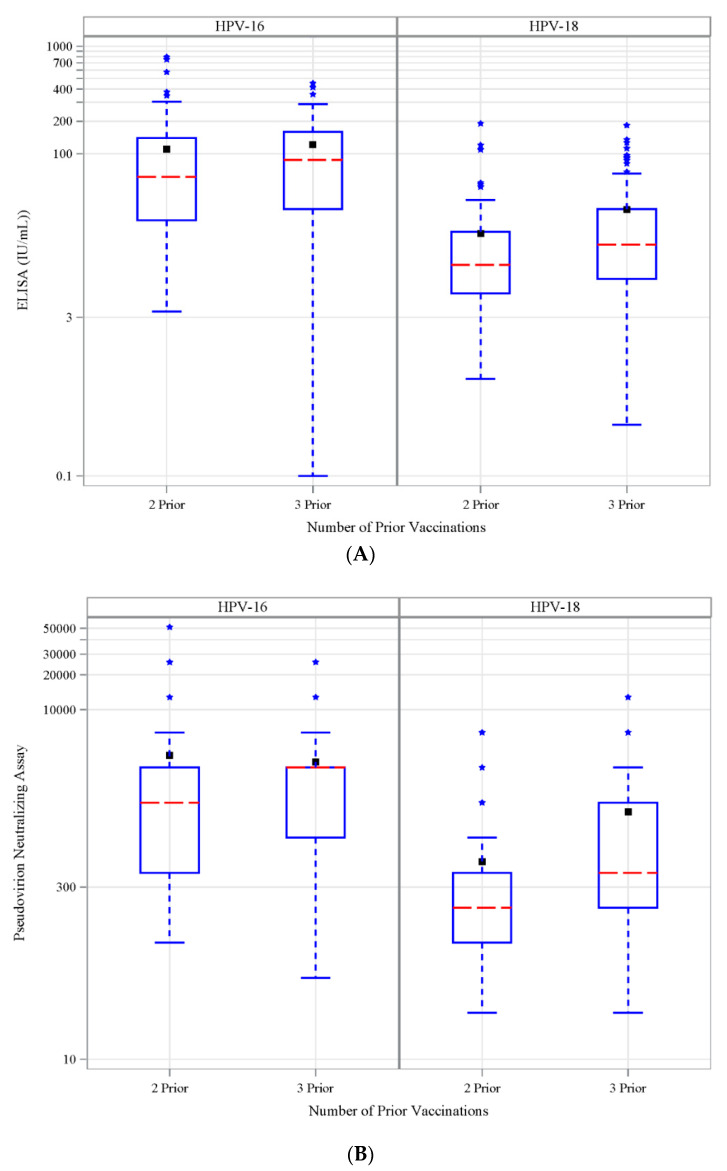
Antibody levels in retrospective cohort. (**A**) HPV-16- (left panel) and HPV-18-specific (right panel) ELISA Ab titers (IU/mL) and (**B**) pseudovirion neutralization titers in retrospective cohort with 2 or 3 doses of prior 4vHPV vaccination.

The elapsed time from the last vaccinations differed significantly (*p* < 0.0001) for the Retro 2 vs. Retro 3 cohorts; the medians were 475 days vs. 1608 days, respectively (Table S4). ELISA and pseudovirion neutralizing Ab titers for both HPV-16 and for HPV-18 were highly correlated in each retro cohort (*p* < 0.0001 for all comparisons, data not shown). ELISA geometric means for HPV-16 and -18 were lower for the Retro 2 cohort compared to Retro 3 (HPV-16 geometric mean 52 vs. 66 IU/mL and HPV-18 geometric mean 10 vs. 15 IU/mL, Retro 2 vs. Retro 3). (Figure 4A and Table S5). A similar pattern was also observed with pseudovirion neutralizing Ab titers (HPV-16 1290 vs. 1815 and HPV-18 228 vs. 488, Retro 2 vs. Retro 3). (Figure 4B; Table S5). These cohort differences were statistically significant only for HPV 18 (both ELISA and neutralizing assays). No significant differences were found for antibody positivity for HPV-16 or HPV-18 in these two cohorts.

### 3.7. HPV Type-Specific MBCs

**Figure 5:**
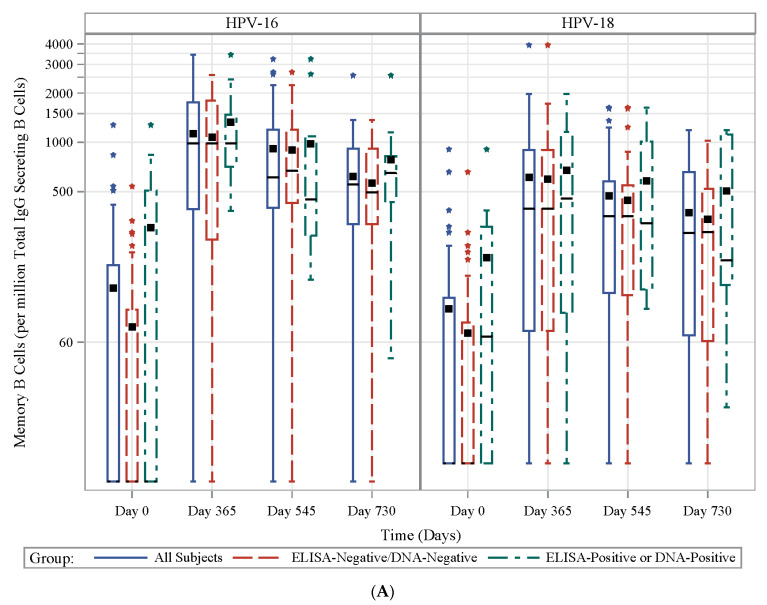

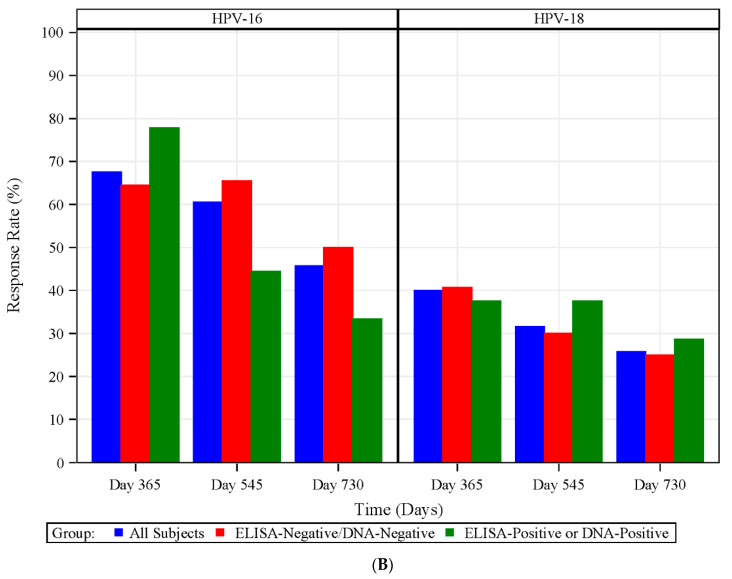
Frequency and proportion of MBC responders induced by 4vHPV. (**A**) Frequency of HPV-16- (left panel) and HPV-18-specific (right panel) specific memory B cells (MBCs) per millions of total IgG-secreting B cells at Days 0, 365, 545 and 730 in the prospective cohort for all (solid blue line), HPV-naïve (dashed red line) and HPV-exposed (dashed green line) subjects; (**B**) proportion of MBC responders (defined as subjects with >455 L-16 B cell or >450 L-18 B cell per millions of total IgG-secreting B cells) to HPV-16- (left panel) and HPV-18- (right panel) in the prospective cohort at Days 365, 454 and 730 as indicated at x-axis for all (blue bar), HPV-naïve (red bar) and HPV-exposed (green bar) subjects.

Overall, the magnitudes of the HPV-specific MBCs (Figure 5A; Table S6) were highest on Day 545 (one year post-last vaccination), but then slightly decreased at Day 730. As expected, baseline MBCs were higher in the HPV-exposed group than in the HPV-naïve group (GM MBCs per 106 total IgG-secreting B cells: 56 vs. 21 for HPV-16 and 65 vs. 24 for HPV-18, exposed vs. naïve, respectively, *p* = 0.10 and *p* = 0.053 for HPV-16 and HPV-18). After vaccination, although MBCs tended to be higher in those previously exposed, no statistically significant difference was observed (data not shown). For all subjects, whether exposed or naïve, 68%, 61%, and 46% responded for HPV-16 and 40%, 32%, and 26% responded for HPV-18 in the MBC assay at Days 365, 545, and 730, respectively (Figure 5B).

**Figure 6:**
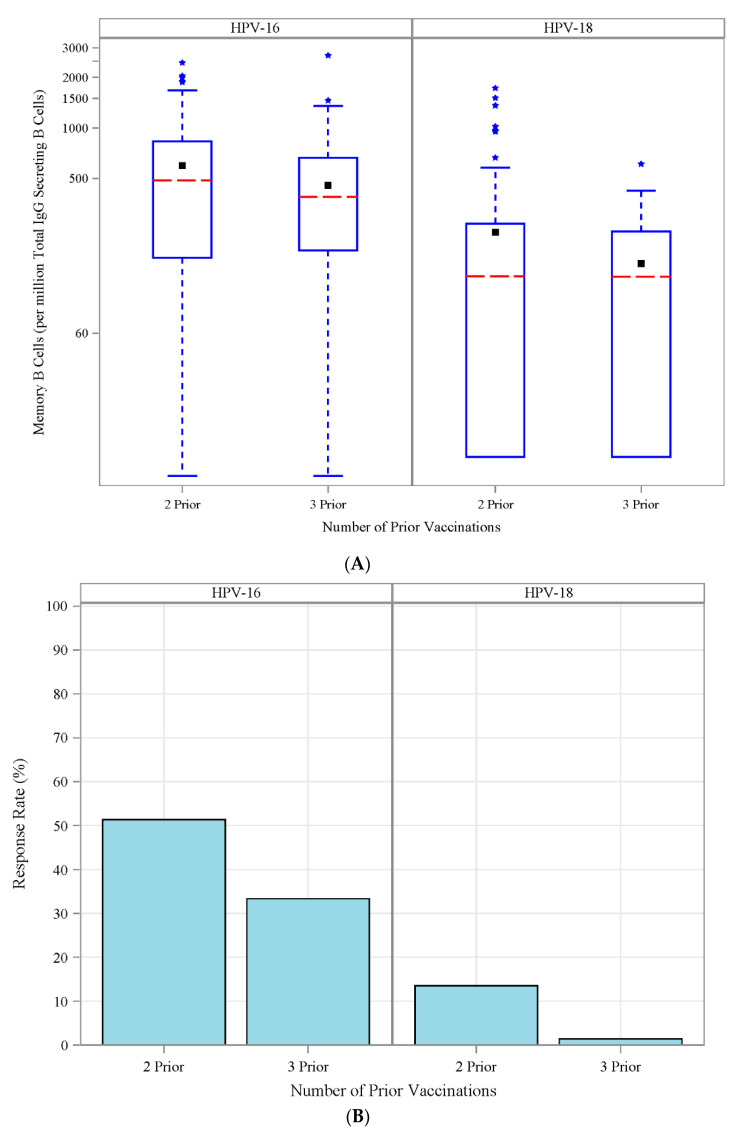
Frequency and proportion of MBC responders in retrospective cohorts. (**A**) Frequency of HPV-16- (left panel) and HPV-18-specific (right panel) specific memory B cells (MBCs) per millions of total IgG-secreting B cells. (**B**) Proportion of MBC responders to HPV-16- (left panel) and HPV-18- (right panel) defined as subjects with detectable (>455 cell/million of total IgG-secreting B cells) or L-18 (>450 cell/million of total IgG-secreting B cells) HPV type-specific memory B cells in retrospective cohort with 2 or 3 doses of prior 4vHPV vaccination.

For the retrospective cohorts, the GM of MBC magnitudes were similar between the cohorts. (306 vs. 241 per 10^6^ total IgG-secreting B cells for HPV-16 and 90 vs. 74 for HPV-18, Retro 2; vs. Retro 3, Figure 6A; Table S7). In contrast, the proportion of subjects in the Retro 2 cohort who responded to HPV-16 and -18 was significantly higher than in Retro 3 (for HPV-16: 38% vs. 24%, for HPV 18: 10% vs. 1%, Figure 6B; Table S7). The proportion of responding subjects was significantly higher for HPV-16 and -18 for the Retro 2 cohort than the Retro 3 cohort (data not shown). These observations are consistent with the fact that the Retro 2 cohort had shorter intervals post-last vaccination than that in the Retro 3 cohort.

## 4. Discussion

Our study comprehensively evaluated the immunological response to three doses of 4vHPV vaccine for two years following initial 4vHPV vaccination, encompassing humoral responses (by ELISA, PBNA and ELISpot) and CD4+ T cell immunity (by ICS and CFSE proliferation assays) in both HPV-naïve and HPV-exposed subjects. In addition, we evaluated the durability of humoral immunity up to seven years postvaccination in two retrospective cohorts.

In the prospective cohort, HPV ELISA antibody levels after two doses were higher among HPV-exposed subjects compared to HPV-naïve subjects; these levels were further boosted by the third dose. Irrespective of HPV type, titers declined one year postdose one but remained detectable two years postvaccination, consistent with other reports [10,19,20,21]. The prospective cohort also demonstrated that vaccine-induced antibody responses were higher than those generated by prior natural exposure. At the two-year time point in the prospective cohort, and many years later in the retrospective cohorts, vaccine-induced antibodies determined by ELISA were still higher than antibodies induced by natural exposure (as measured by baseline values in the HPV-exposed group in the prospective cohort). Naturally acquired antibodies to HPV-16, and to a lesser extent HPV-18, are associated with some reduced risk of subsequent infection and cervical abnormalities associated with the same HPV type [2,21]. The retrospective cohorts demonstrated higher antibody titers in the Retro 3 cohort (median elapsed time almost five years after the third vaccine dose) compared to the Retro 2 cohort (median elapsed time 1.25 years after the second vaccine dose). This somewhat counterintuitive observation was likely due to the number of doses in each cohort and timing of the final (booster) dose (one month vs. six months after the first vaccine dose in the Retro 2 vs. Retro 3 cohorts, respectively) [2,22]. It is now known that a longer rest interval between the priming and boosting vaccine doses may result in a more robust plasmablast response and higher antibody titers after boost [23].

In our prospective cohort, higher baseline MBC magnitudes were detected in HPV-exposed subjects compared to HPV-naïve subjects, but these magnitudes were lower than those observed in both Retro 2 and Retro 3 cohorts, indicating that vaccination could induce more durable MBC response than natural exposure. A prior study showed that antibodies cloned from naturally elicited MBCs were generally non-neutralizing, whereas those isolated following vaccination were neutralizing [23]. Our study compared the MBC responses in subjects receiving 2 versus 3 doses of 4vHPV. The higher circulating MBCs in the Retro 2 compared to the Retro 3 cohort were likely due to the shorter postvaccination interval in the Retro 2 cohort (median 475 days) compared to the Retro 3 cohort (median 1608 days). The frequency of MBCs after a two-dose vaccination schedule (zero and six months) vs. three-dose schedule (zero, one and six months) over the same interval should be studied as the two-dose schedule is now used for individuals younger than 15 years of age [24]. Though both antibody and MBC postvaccination responses were higher for HPV-16 compared to HPV-18 consistent with other studies [25], the type-specific CD4+ T cell responses were similar in assays using L1 peptides for stimulation [10].

Although induction of antigen-specific memory B cells, a process in which CD4+ T cells are essential, is thought to be important for long-term vaccine-induced protection [25,26], the role of the cell-mediated immune response in the control of HPV exposure is not well established. In general, the CD4+ T cell magnitudes and kinetics measured by ICS in the prospective cohort were consistent with prior findings [10,20]. Interestingly, the ICS response was significantly higher after both the second and the third doses than at baseline, but there was no statistically significant difference between two and three doses, suggesting that two rather than three doses are sufficient to induce an adequate CD4+ T cell response. Unique aspects of our study include identifying detectable cytokine-producing, HPV type-specific CD4+ T cell responses in HPV-exposed but unvaccinated subjects. Another novel finding was the demonstration of a correlation between CD4+ T cell magnitudes seven days postvaccination and antibody levels after second and third vaccinations. A detailed analysis of the association between circulating T follicular helper cells (PD1^+^ICOS^+^ Tfh1-like subset) with antibody and MBC responses using HPV vaccines as a successful model would be worth conducting on a larger scale [27,28] to better understand the interplay between B and T cell immune responses.

A literature search identified only a single published study of lymphocyte proliferation following HPV vaccination, with assays performed only one month post-second and third doses [28]. In our prospective cohort, we detected CD4+ T cell proliferative responses two years postvaccination with higher responses at later time points (in contrast to the ICS assay where magnitudes decreased over time). For subjects in the Retro 3 cohort, CD4+ T cell proliferation was readily detected at a median of five years following the last 4vHPV vaccine dose. This strong, late CD4+ T cell proliferative potential, upon stimulation by HPV peptides, is an interesting finding and is very desirable for a vaccine that needs to provide long-term protection. This finding indicates that the 4vHPV vaccine-induced high-quality memory CD4+ T cells [5].

A recent publication showed that in a high vaccine coverage setting, one dose had similar effectiveness as two or three doses in preventing high-grade disease cervical disease [29]. We did not collect blood samples post-first vaccine dose in the prospective cohort, so we are not able to evaluate responses early in the series. Our study had several other limitations. We did not study the currently available nine valent vaccine. The elapsed time after either the first or last 4vHPV vaccine doses in the two retrospective cohorts were quite different, making immune response comparisons between the two retro cohorts difficult to interpret. We did not study a two-dose schedule with a six-month interval as is now recommended for those initiating vaccination at age 15 or younger.

## 5. Conclusions

The study sheds new light on the crucial role of the B and T cell responses and their importance in the duration of immunity after 4vHPV vaccination with no need for a booster per ACIP [24]. The results demonstrated good immunogenicity over a two-year follow-up period, and enhanced serological and cellular responses were observed in those who already had prior exposure.

## Figures and Tables

**Table 1:** Demographic and baseline human papillomavirus virus (HPV) exposure characteristics of subjects in prospective and two retrospective cohorts.

Characteristic Measured	Prospective (*n* = 47)	Retrospective with 2 Prior HPV Vaccinations (*n* = 78)	Retrospective with 3 Prior HPV Vaccinations (*n* = 78)	All Cohorts (*n* = 203)
Gender, *n* (% ^1^)	-	-	-	-
Female	47 (100)	78 (100)	78 (100)	203 (100)
Ethnicity, *n* (% ^1^)	-	-	-	-
Non-Hispanic or Non-Latino	47 (100)	72 (92)	75 (96)	194 (96)
Hispanic or Latino	-	6 (8)	3 (4)	9 (4)
Race-*n* (% ^1^)	-	-	-	-
American Indian/Alaskan Native	1 (2)	-	-	1 (0)
Asian	7 (15)	17 (22)	3 (4)	27 (13)
Black or African American	14 (30)	16 (21)	8 (10)	38 (19)
White	24 (51)	44 (56)	64 (82)	132 (65)
Multi-Racial	-	-	3 (4)	3 (1)
Other/Unknown	1 (2)	1 (1)	-	2 (1)
Age (years)	-	-	-	-
Mean (SD)	24 (2)	25 (3)	25 (3)	25 (3)
Median	24	25	24	25
Min, Max	18, 26	19, 30	18, 30	18, 30
Baseline HPV exposure	-	-		
ELISA-negative and DNA-negative	-			
HPV-16	36 (77)			
HPV-18	37 (79)			
ELISA-Positive or DNA-Positive	-			
HPV-16	11 (23)			
HPV-18	10 (21)			

^1^ Denominator for percentages is the number of subjects enrolled for each cohort. *n*: The total number of subjects.

**Table 2:** Comparison of antibody responses in the HPV-exposed group with HPV-naïve in the prospective cohort.

Timepoint	HPV-16			HPV-18		
Days Postvaccination	ELISA− and PCR−	ELISA+ Or PCR+	*p*-Value	ELISA− and PCR−	ELISA+ or PCR+	*p*-Value
Day 0	0	10	<0.0001	0	4	<0.0001
Day 67	165	498	0.0330	39	165	0.0060
Day 187	208	442	0.0350	74	121	0.3080
Day 365	111	243	0.0290	28	79	0.0340
Day 545	65	173	0.0120	14	51	0.0230
Day 730	51	143	0.0120	11	32	0.0370

**Table 3:** Correlation between CD4+ T Cell and antibody responses for HPV-16 and HPV-18 in the prospective cohort.

Timepoint			Antibody Titer by Study Time Point				
Type	Cytokine Study Time Point	Statistics	Day 67 (Dose 2 + 7 Days)	Day 187 (Dose 3 + 7 Days)	Day 365	Day 545	Day 730
HPV-16	Day 67 (Day 7 postdose 2)	Number of Subjects	44	40	39	37	34
		Correlation Statistic ^1^	0.366	0.568	0.529	0.470	0.479
		*p*-value ^2^	0.0146	0.0001	0.0005	0.0033	0.0041
	Day 187 (Day 7 postdose 3)	Number of Subjects	37	37	36	34	32
		Correlation Statistic ^1^	0.378	0.525	0.530	0.379	0.400
		*p*-value ^2^	0.0211	0.0008	0.0009	0.0269	0.0232
HPV-18	Day 67 (Day 7 postdose 2)	Number of Subjects	44	40	39	37	34
		Correlation Statistic ^1^	0.416	0.334	0.415	0.376	0.275
		*p*-value ^2^	0.0050	0.0352	0.0087	0.0217	0.1150
	Day 187 (Day 7 postdose 3)	Number of Subjects	38	38	37	35	33
		Correlation Statistic ^1^	0.400	0.383	0.311	0.287	0.210
		*p*-value ^2^	0.0128	0.0175	0.0607	0.0946	0.2395

Subjects with both values at a time point are summarized. ^1^ Spearman Rank Correlation Coefficient. ^2^ *p*-value is testing the null hypothesis of zero correlation.

## Supplementary Materials

The following are available online at https://www.mdpi.com/2076-393X/8/3/348/s1, Table S1: Number of IL-2, IL-4, IL-21, and/or INF-γ Positive Cells per Million CD4 T Cells by Previous Exposure to HPV, Table S2: Baseline and Postvaccination ELISA Results in Prospective Cohort Stratified by HPV Exposure Status, Table S3: Percentage of CFSE^low^ CD4 T cells (%) by Previous Exposure to HPV, Table S4: Comparison of Elapsed Time in Days from Last Vaccination at Enrollment in Retrospective Cohorts, Table S5: Antibody Levels in Retrospective Cohorts, Table S6: Number of HPV + Memory B cells Per Million of Total-IgG-secreting B cells by Previous Exposure to HPV, Table S7: Number of HPV + Memory B Cells per Million of Total IgG Secreting B cells in Retrospective Cohorts.

## Appendix A

### Appendix A.1. Multiplexed Enzyme-Linked Immunosorbent Assay

Antibody responses to HPV16 and 18 were measured using multiplex M4ELISA [13]. Briefly, type-specific HPV L1 + L2 virus-like particles were used as antigen to detect IgG responses in a direct ELISA using the Meso Scale Discovery platform. Test and reference sera were serially diluted to determine antibody titer using the parallel line method [14]. Seropositivity was determined using cut-off value as equal to or above the 99th percentile of Johnson-Su probability distribution of children’s sera signal tested at 1:100 (gift from Dr. Joakim Dillner, Lund University, Sweden). Titers are reported in International Units/mL for both HPV types.

### Appendix A.2. Intracellular Cytokine Assay

ICS assays were performed as described previously [30].Cryopreserved PBMCs were thawed and rested overnight, and then incubated for 6 h at 37 °C with virus peptide pools at final concentrations of 2 μg/mL of each peptide in the presence of CD28 and CD49d (BD Biosciences, 340957 and 340976). Negative control samples were left unstimulated, and positive control samples were treated with *Staphylococcus* enterotoxin B (Sigma-Aldrich, St. Louis, MO, USA) at a final concentration of 1 μg/mL. mL penicillin, 0.1 mg/mL streptomycin, 0.3 mg/mL glutamine, and 10% FBS). Cells were cultured for 2 h at 37 °C, and then a cocktail containing brefeldin A and monensin was added (eBioscience,004980-93, San Diego, CA, USA), followed by a further 4 h of culture. Cells were washed with 1xPBS; surface stained with Aqua live/dead stain L423102 (Biolegend, San Diego, CA, USA), and then fixed and permeabilized using a Cytofix/Cytoperm kit (BD Biosciences; 554722, Franklin Lakes, NJ, USA). The cells were then stained with following fluorescence conjugated antibodies: CD3(SP34-2), CD4(L200), IL-2 (MQ1-17H12, #554567) and TNF-α (MAB11, #550679) from BD; and IFN-g (4S.B3, #47-731942) and IL-21(2A3-N2) from eBioSciences. After the cells were washed data were collected on an LSRII Fortessa instrument (BD Biosciences, Franklin Lakes, NJ, USA). Compensation was performed using tubes of CompBeads (BD Biosciences, Franklin Lakes, NJ, USA#51-90-9001229) individually stained with each fluorophore and compensation matrices were calculated with FACSdiva. Data were analyzed using FlowJo software version 9 (Tree Star, Ashland, OR, USA).

### Appendix A.3. Memory B-Cell (MBC) ELISpot Assay

MBC frequencies were evaluated using a previously described B cell ELISpot assay [31]. Thawed PBMCs were plated in 24-well dishes at 5 × 10^5^ cells/well in R-10 medium supplemented with IL-2 and R488 (CTL-hBPOLYS-200, CTL). Eight wells were cultured per individual for six days. For total IgG-secreting and HPV-specific MBCs, ninety-six–well filter plates (Millipore, Burlington, MA, USA) were coated with VLP antigens as described above (same antigen as in the ELISA assay) at 2 ug/mL or anti-Human IgG (10 μg/mL). The plates were left to adsorb overnight at 4 °C. Plates were then washed four times in PBS and incubated in RPMI medium with 10% (vol/vol) FBS for 30 min at 37 °C. RPMI was removed, and stimulated cells were suspended in RPMI with 10% FBS were placed in each well, with threefold dilutions down the plate. Plates were incubated at 37 °C overnight. The following day, plates were washed four times in PBST and incubated for 2 h at room temperature with biotinylated anti-human IgG (MT78/145; Mabtech) at 1 μg/mL diluted in PBST with 1% FBS. Plates were washed four times in PBST and incubated for 1 h at room temperature with streptavidin-HRP (Mabtech, Nacka Strand, Sweden) diluted 1:1000 in PBST with 1% FBS. Plates were washed three times each in PBST and PBS, and then incubated with TMB (3,3′,5,5′-tet- ramethylbenzidine) substrate for 5–10 min until spot development. Developed plates were scanned and analyzed using an automated ELISpot counter (Cellular Technologies, Shaker Heights, OH, USA). Data are presented as the percentage of influenza virus-specific immunoglobulin G (IgG)-secreting cells among total IgG-secreting cells.

### Appendix A.4. Carboxyfluorescein Succinimidyl Ester (CFSE) Assay

CFSE dilution assays were performed as described previously [32]. Briefly, PBMC were prestained with CFSE and approximately 1 × 10^6^ cells were stimulated in 24-well plates in a volume of 1 mL in RPMI containing 10% human serum at 37 °C under 5% CO_2_ for six days. Cells were stimulated with pooled peptides at a concentration of 2 μg/mL of each peptide as described above. Unstimulated cells served as negative controls. At the end of six days in culture, cells were harvested and stained on the surface with following fluorescence conjugated antibodies: CD3(SP34-2), CD4(L200), CD8 (RPA-T8) and samples were acquired on LSRII.

### Appendix A.5. Statistical Analysis

The primary hypothesis of this study was that the three-dose series of HPV4 vaccine led to long-term immune memory mediated by T and B Lymphocytes. It was assumed that HPV “naïve” subjects would have no memory B cells prior to vaccination. We tested whether subjects had a positive memory B cell response after the third dose of vaccine using a one-sample t-test at the one-sided 0.025 significance level. The significance level was selected to correspond to a 95% confidence interval (CI) for the mean level of memory B cell response postvaccination based on the t-statistic. Similar clinical studies have shown that 1/3 of subjects in this age range are positive for HPV DNA or seropositive for HPV antibodies. Therefore, we estimated that 27 women were needed to complete the entire two-year protocol for the prospective group, so 45 women were enrolled for the initial visit. The retrospective cohorts consisted of women having already received a complete or partial vaccine series. We assumed that 80% of women in these cohorts would have neutralizing antibodies. With a sample size of 78, the average exact (Clopper–Pearson) CI for the percentage of responders extended from 69% to 88%, and the study had 80% power to rule out a response percentage below 67%.
