# Supplementary material for: Duration of Cellular and Humoral Responses after Quadrivalent Human Papillomavirus Vaccination in Healthy Female Adults with or without Prior Type 16 and/or 18 Exposure
Source: Vaccines (Basel). 2020 Jun 30;8(3):348. doi: 10.3390/vaccines8030348 (PMC7563427; doi:10.3390/vaccines8030348)
Supplement: Supplementary file 1 [file vaccines-08-00348-s001.pdf]

Table S1. Number of IL-2, IL-4, IL-21, and/or INF-γ Positive Cells per Million CD4 T Cells by Previous Exposure to HPV.

	HPV-16			HPV-18		
**Measurement at Study Timepoint**	**ELISA-Negative and DNA-Negative**	**ELISA-Positive or DNA-Positive**	**All Subjects**	**ELISA-Negative and DNA-Negative**	**ELISA-Positive or DNA-Positive**	**All Subjects**
**Day 0**						
N	33	11	44	34	10	44
Min, Max	0, 291	0, 1888	0, 1888	0, 416	0, 2329	0, 2329
Geometric Mean (GSD)	59 (1)	94 (3)	66 (2)	33 (2)	76 (6)	40 (3)
**Day 67**						
N	33	11	44	34	10	44
Min, Max	108, 2746	113, 3866	108, 3866	53, 3211	296, 3371	53, 3371
Geometric Mean (GSD)	672 (2)	873 (3)	718 (2)	588 (2)	1137(2)	683 (2)
95% CI of Geometric Mean [1]	498, 908	416,1832	544, 947	436, 793	608, 2126	521, 897
P-Value[2]	0.0197	0.0314	0.0003	< 0.0001	0.0002	< 0.0001
Responders (%)[3]	30 (91%)	9 (82%)	39 (89%)	26 (77%)	9 (90%)	35 (80%)
95% CI for Responders (%)[4]	76%, 98%	48%, 97%	75%, 96%	59%, 89%	56%, 100%	65%, 90%
**Day 187**						
N	29	8	37[5]	30	8	38
Min, Max	233, 3718	140, 4557	140, 4557	173, 2690	234, 2126	173, 2690
Geometric Mean (GSD)	972 (2)	1021 (3)	983 (2)	841(2)	972(3)	867 (2)
95% CI of Geometric Mean[1]	751, 1259	366, 2845	751, 1286	662, 1069	455, 2077	690, 1089
P-Value[2]	0.0002	0.0459	< 0.0001	< 0.0001	0.0008	< 0.0001
Responders (%)[3]	28 (97%)	6 (75%)	34 (92%)	27 (90%)	6 (75%)	33 (87%)
95% CI for Responders (%)[4]	82%, 100%	35%, 97%	78%, 98%	73%, 98%	35%, 97%	72%, 96%

Note: N = Number of subjects in the prospective cohort. GSD=Geometric Standard Deviation; CI=Confidence Interval.

[1]CI is estimated using a two-sided Normal Approximation Method.

[2]Based on a regression analysis with baseline in the model using a 0.05% significance level testing that the slope equals one.

[3]A responder was defined as subjects whose value is greater than baseline value and with >180 HPV-16+ or >296 HPV-18+ CD4 T cells per million of total CD4 T cells.

[4]CI is estimated using a two-sided Exact (Clopper-Pearson) Method .

[5]HPV-16 test in one of the samples was not qualified for data analysis.

Table S2. Baseline and Post-Vaccination ELISA Results in Prospective Cohort Stratified by HPV Exposure Status.

	Antibody Levels by ELISA (IU/mL))					
	HPV-16			HPV-18		
	**ELISA-Negative and DNA-Negative**	**ELISA-Positive or DNA-Positive**	**All Subjects**	**ELISA-Negative and DNA-Negative**	**ELISA-Positive or DNA-Positive**	**All Subjects**
**Day 0 (pre dose 1)**						
N	36	11	47	37	10	47
Min, Max	0.0, 1.3	0.1, 354.0	0.0, 354.0	0.0, 0.9	1.6, 12.8	0.0, 12.8
Geometric Mean (GSD)	0.2 (2.8)	9.80 (9.0)	0.4 (9.2)	0.2 (2.8)	3.9 (2.2)	0.3 (5.2)

**Day 67 (day 7 post dose 2)**						
N	34	11	45	35	10	45
Min, Max	7.4, 2650.0	10.4, 4320.0	7.4, 4320.0	1.7, 788.2	21.5, 823.5	1.7, 823.5
Geometric Mean (GSD)	165.0 (3.6)	499.0 (6.7)	216.3(4.5)	39.2 (4.1)	165.5(3.4)	54.0 (4.4)
95% CI of Geometric Mean [1]	105.9, 257.0	138.2, 1801.5	137.5, 340.1	24.2, 63.7	68.3, 400.7	34.6, 84.5
P-Value [2]	0.0004	0.0926	< 0.0001	0.0007	0.4052	< 0.0001
Responders (%) [3]	34 (100.0%)	11 (100.0%)	45 (100.0%)	35 (100.0%)	10 (100.0%)	45 (100.0%)
95% CI for Responders (%)[4]	89.7%, 100.0%	71.5%, 100.0%	92.1%, 100.0%	90.0%, 100.0%	69.2%, 100.0%	92.1%, 100.0%
**Day 187 (day 7 post dose 3)**						
N	32	9	41	32	9	41
Min, Max	20.1, 780.0	75.8, 2840.0	20.1, 2840.0	2.5, 947.1	23.7, 444.7	2.5, 947.1
Geometric Mean (GSD)	207.8 (2.3)	442.0 (3.4)	245.3 (2.6)	74.3 (3.8)	120.8 (2.4)	82.7 (3.5)
95% CI of Geometric Mean[1]	154.8, 279.0	172.1, 1135.5	181.2,331.8	46.0, 119.9	61.9, 235.8	55.8, 122.6
P-Value [2]	< 0.0001	0.0091	< 0.0001	0.0014	0.1439	< 0.0001
Responders (%) [3]	32 (100.0%)	9 (100.0%)	41 (100.0%)	32 (100.0%)	9 (100.0%)	41 (100.0%)
95% CI for Responders (%)[4]	89.1%, 100.0%	66.4%, 100.0%	91.4%, 100.0%	89.1%, 100.0%	66.4%, 100.0%	91.4%, 100.0%
**Day 365**						
N	31	9	40	32	8	40
Min, Max	20.5, 604.0	24.0, 1400.0	20.5, 1400.0	2.4, 399.4	13.8, 374.1	2.4, 399.4
Geometric Mean (GSD)	110.8 (2.2)	243.3 (3.7)	132.3 (2.6)	27.5 (3.5)	78.8 (2.8)	34.0 (3.6)
95% CI of Geometric Mean[1]	83.5, 147.1	88.8, 666.7	97.3, 179.	17.5, 43.2	33.8, 183.6	22.7, 51.0
P-Value [2]	< 0.0001	0.0148	< 0.0001	0.0001	0.1631	< 0.0001
Responders (%) [3]	31 (100.0%)	9 (100.0%)	40 (100.0%)	32 (100.0%)	8 (100.0%)	40 (100.0%)
95% CI for Responders (%)[4]	88.8%, 100.0%	66.4%, 100.0%	91.19%, 100.0%	89.1%, 100.0%	63.1%, 100.0%	91.2%, 100.0%
**Day 545**						
N	29	9	38	30	8	38
Min, Max	11.2, 409.0	14.4, 779.0	11.2, 779.0	0.0, 201.8	11.4, 327.7	0.0, 327.7
Geometric Mean (GSD)	65.0 (2.3)	172.5 (3.9)	81.6 (2.8)	13.7 (5.0)	50.9 (2.7)	18.1 (4.9)
95% CI of Geometric Mean[1]	47.7, 88.5	60.5, 492.2	58.2, 115.2	7.5 , 25.0	22.3, 116.3	10.8, 30.4
P-Value [2]	< 0.0001	0.0183	< 0.0001	0.0078	0.2333	< 0.0001
Responders (%) [3]	29 (100.0%)	9 (100.0%)	38 (100.0%)	29 (96.7%)	8 (100.0%)	37 (97.4%)
95% CI for Responders (%)[4]	88.1%, 100.0%	66.4%, 100.0%	90.8%, 100.0%	82.8%, 99.9%	63.0%, 100.0%	86.2%, 99.9%
**Day 730**						
N	26	9	35	28	7	35
Min, Max	17.3, 370.0	12.1, 606.0	12.1, 606.0	0.8, 155.9	8.1, 68.8	0.8, 155.9
Geometric Mean (GSD)	50.7 (2.3)	142.7 (4.2)	66.2 (3.0)	10.9 (3.5)	32.2 (2.2)	13.5 (3.5)
95% CI of Geometric Mean[1]	36.4, 70.6	47.5, 428.7	45.5, 96.2	6.7, 17.7	15.6, 66.4	8.8, 20.7

P-Value [2]	< 0.0001	0.0356	< 0.0001	0.0010	0.0009	< 0.0001
Responders (%) [3]	26 (100.0%)	9 (100.0%)	35 (100.0%)	27 (96.4%)	7 (100.0%)	34 (97.1%)
95% CI for Responders (%)[4]	86.8%, 100.0%	66.4%, 100.0%	90.0 %, 100.0%	81.7%, 99.9%	59.0 %, 100.0%	85.1%, 99.9%

Note: N = Number of subjects in the prospective cohort. GSD=Geometric Standard Deviation; CI=Confidence Interval.[1] CI is estimated using a two-sided Normal Approximation Method [2] Based on a regression analysis with baseline in the model using a 0.05% significance level testing that the slope equals one. [3] A responder was defined as seropositive by the laboratory. The denominator for percentages is based on the total number of subjects (n) with a result at the visit being summarized. [4] CI is estimated using a two-sided Exact (Clopper-Pearson) Method.

Table S3. Percentage of CFSE^{low} CD4 T cells (%) by Previous Exposure to HPV.

	**HPV-16**			**HPV-18**		
	**ELISA-Negative and DNA-Negative**	**ELISA-Positive or DNA-Positive**	**All Subjects**	**ELISA-Negative and DNA-Negative**	**ELISA-Positive or DNA-Positive**	**All Subjects**
**Day 0**						
N	35	10	45	36	9	45
Min, Max	0.00, 1.11	0.00, 0.54	0.00, 1.11	0.00, 1.24	0.00, 1.18	0.00, 1.24
Geometric Mean (GSD)	0.00 (4.09)	0.00 (3.73)	0.00 (3.97)	0.00 (8.69)	0.00 (18.23)	0.00 (10.86)
**Day 365**						
N	31	9	40	32	8	40
Min, Max	0.00, 14.73	0.00, 5.57	0.00, 14.73	0.00, 12.33	0.00, 4.43	0.00, 12.33
Geometric Mean (GSD)	0.85 (5.22)	0.32 (5.59)	0.68 (5.46)	0.59 (5.43)	0.44 (12.59)	0.56 (6.39)
95% CI of Geometric Mean[1]	0.47, 1.57	0.08, 1.19	0.38, 1.18	0.32, 1.09	0.05, 3.64	0.31, 1.01
P-Value [2]	0.0250	0.0216	0.0011	0.0002	0.0079	< 0.0001
Responders (%) [3]	25 (80.6%)	6 (66.7%)	31 (77.5%)	30 (93.8%)	5 (62.5%)	35 (87.5%)
95% CI for Responders (%)[4]	62.5%, 92.6%	29.9 %, 92.5 %	61.6%, 89.2%	79.2%, 99.2 %	24.5%, 91.5%	73.2%, 95.8%
**Day 545**						
N	28	9	37	29	8	37
Min, Max	0.00, 16.15	0.04, 7.61	0.00, 16.15	0.02, 16.65	0.00, 7.77	0.00, 16.65
Geometric Mean (GSD)	1.126 (6.14)	0.57 (5.42)	0.95 (5.97)	0.75 (6.30)	0.51 (15.23)	0.69 (7.58)
95% CI of Geometric Mean[1]	0.56, 2.28	0.156, 2.09	0.53, 1.73	0.37 1.50	0.052, 4.95	0.35, 1.35
P-Value [2]	0.1435	0.4711	0.0920	0.0018	0.3280	0.0002
Responders (%) [3]	25 (89.3%)	7 (77.8%)	32 (86.5%)	28 (96.6%)	4 (50.0%)	32 (86.5%)
95% CI for Responders (%)[4]	71.8%, 97.7%	40.0%, 97.2%	71.2 %, 95.5%	82.2%, 99.9%	15.7%, 84.3%	71.2 %, 95.5%
**Day 730**						
N	26	9	35	28	7	35
Min, Max	0.08, 9.48	0.10, 8.73	0.08, 9.48	0.06, 4.77	0.16, 10.48	0.06, 10.48
Geometric Mean (GSD)	1.39 (3.22)	0.65 (4.76)	1.14 (3.67)	0.77 (3.28)	1.97 (3.77)	0.93 (3.51)
95% CI of Geometric Mean[1]	0.87 2.23	0.20, 2.15	0.73, 1.79	0.48, 1.21	0.58, 6.73	0.60, 1.43
P-Value [2]	0.0010	0.1949	0.0006	< 0.0001	0.0241	< 0.0001
Responders (%) [3]	24 (92.3%)	7 (77.8%)	31 (88.6%)	26 (92.9%)	4 (57.1%)	30 (85.7%)
95% CI for Responders (%)[4]	74.9%, 99.1%	40.0%, 97.2%	73.3%, 96.8 %	76.5 %, 99.1%	18.4%, 90.1%	69.7%, 95.2%

Note: N = Number of subjects in the prospective cohort. GSD=Geometric Standard Deviation; CI=Confidence Interval. [1] CI is estimated using a two-sided Normal Approximation Method. [2] Based on a regression analysis with baseline in the model using a 0.05% significance level testing that the slope equals one. [3] A responder was defined as having a result two times above the baseline value. The denominator for percentages is based on the total number of subjects (n) with a result at the visit being summarized. [4] CI is estimated using a two-sided Exact (Clopper-Pearson) Method.

Table S4: Comparison of Elapsed Time in Days from Last Vaccination at Enrollment in Retrospective Cohorts.

Statistics	Retrospective With 2 Prior HPV Vaccinations (N=74)	Retrospective With 3 Prior HPV Vaccinations (N=77)	Difference 3 Prior versus 2 Prior
**Mean (SD)**	748 (619)	1581 (222)	833 (461)
**Median**	471 (16 months)	1608(54 months)	
**Min, Max**	181, 2419	1100, 1986	
**95% CI of Mean**			684,980 [1]
**P-Value**			<0.0001 [2]

Note: N=subjects with samples can be tested. [1] 95% CI was calculated using a two-sided test using Normal Approximation Method. [2] P-value is based on a two-sample t-test using a 2-sided 0.05% significance level.

***Table S5*:** Antibody Levels in Retrospective Cohorts.

	ELISA (IU/mL)			Pseudovirion Neutralizing Assay (1/dilution)		
	**Retro 2 (N=74)**	**Retro 3 (N=77)**	**Difference 3 Prior - 2 Prior**	**Retro 2 (N=74)**	**Retro 3 (N=77)**	**Difference 3 Prior - 2 Prior**
**HPV-16**						
Min, Max	3.4, 794.0	0.1, 1280.0	N/A	100.0, 51200.0	0.0, 25600.0	N/A
Geometric Mean (GSD)	52.5 (3.7)	65.9 (3.8)	1.3 (3.7) [6]	1289.9 (4.2)	1814.8 (3.8)	1.4 (4.0) [6]
95% CI of Geometric Mean [1]	38.9, 70.9	48.7, 89.1	0.8, 1.9[6]	922.6, 1803.3	1340., 2456.5	0.9, 2.2[6]
**P-Value [2]**			**0.2885**			**0.1335**
Seropositive (%) [3]	74 (100.0%)	76 (98.7%)	-1.3% [7]	74 (100.0%)	75 (97.4%)	-2.6% [7]
95% CI for Seropositive (%) [4]	95.1 %, 100.0%	93.0%, 100.0%	-3.8 %, 1.2 % [7]	95.1 %, 100.0 %	90.9 %, 99.7%	-6.2%, 1.0% [7]
**P-Value [5]**			**0.3253**			**0.1628**
**HPV-18**						
Min, Max	0.8, 190.6	0.3, 183.5	N/A	0.0, 6400.0	0.0, 12800.0	N/A
Geometric Mean (GSD)	9.7 (3.0)	15.5 (3.4)	1.6 (3.2) [6]	228.0 (3.3)	487.6 (4.4)	2.1 (3.9) [6]
95% CI of Geometric Mean [1]	7.5, 12.5	11.7, 20.4	1., 2.3[6]	172.6, 301.2	347.9, 683.3	1.4, 3.3[6]
**P-Value [2]**			**0.0142**			**0.0007**
Seropositive (%) [3]	69 (93.2%)	73 (94.8%)	1.6% [7]	68 (91.9%)	73 (94.8%)	2.9% [7]
95% CI for Seropositive (%) [4]	84.9%, 97.8%	87.2%, 98.6%	-6.0%, 9.1% [7]	83.2%, 97.0%	87.2%, 98.6%	-5.0%, 10.9% [7]
**P-Value [5]**			**0.6853**			**0.4717**

Note: N=Numbers of subjects with samples can be tested. GSD=Geometric Standard Deviation; CI=Confidence Interval. [1] Confidence Interval (CI) is estimated using a two-sided Normal Approximation Method. [2] P-value is based on a two-sample t-test using a 2-sided 0.05% significance level. [3] A responder was defined as seropositive by the laboratory. The denominator for percentages is based on the total number of subjects (n) with a result at the visit being summarized. [4] CI is estimated using a two-sided Exact (Clopper-Pearson) Method. [5] P-value is based on a two-sample Chi-square test using a 2-sided 0.05% significance level. [6] Difference is the relative difference. A value of one means the response after two doses is the same as the response after three doses. [7] Difference is the absolute difference.

Table S6: Number of HPV + Memory B cells Per Million of Total-IgG secreting B cells by Previous Exposure to HPV.

	Memory B Cells (Number of HPV specific memory B cells/per million of total IgG secreting cells)	
	**HPV-16**	**HPV-18**

	ELISA-Negative and DNA-Negative	ELISA-Positive or DNA-Positive	All Subjects	ELISA-Negative and DNA-Negative	ELISA-Positive or DNA-Positive	All Subjects
**Day 0**						
N	35	11	46	36	10	46
Min, Max	0, 538	0, 1276	0, 1276	0, 658	0, 906	0, 906
Geometric Mean (GSD)	21 (4)	56 (9)	27 (6)	24 (4)	65 (6)	30 (4)
**Day 365**						
N	31	9	40	32	8	40
Min, Max	0, 2574	382, 3430	0, 3430	0, 3931	0, 1974	0, 3931
Geometric Mean (GSD)	447 (8)	1068 (2)	544(6)	209 (6)	274 (6)	221(6)
95% CI of Geometric Mean[1]	217, 921	631,1806	306, 965	108, 405	63, 1199	124, 393
P-Value [2]	0.0010	< 0.0001	< 0.0001	0.0006	0.0780	< 0.0001
Responders (%) [3]	20 (64.5%)	7 (77.8%)	27 (67.5%)	13 (40.6%)	3 (37.5%)	16 (40.0%)
95% CI for Responders (%)[4]	45.37%, 80.77%	39.99%, 97.19%	50.87%, 81.43%	23.70%, 59.36%	8.52%, 75.51%	24.86%, 56.67%
**Day 545**						
N	29	9	38	30	8	38
Min, Max	0, 2684	145, 3228	0, 3228	0, 1629	96, 1630	0, 1630
Geometric Mean (GSD)	610 (3)	562 (3)	599 (3)	238 (4)	339 (3)	256(4)
95% CI of Geometric Mean[1]	397, 938	242, 1305	416, 862	142, 399	131, 878	165, 397
P-Value [2]	< 0.0001	0.0021	< 0.0001	< 0.0001	0.0132	< 0.0001
Responders (%) [3]	19 (65.5%)	4 (44.4%)	23 (60.5%)	9 (30.0%)	3 (37.5%)	12 (31.6%)
95% CI for Responders (%)[4]	45. 7%, 82.1%	13.7%, 78.8 %	43.4%, 76.0%	14.7%, 49.4%	8.5%, 75.5%	17.5%, 48.7%
**Day 730**						
N	26	9	35	28	7	35
Min, Max	0, 1374	48, 2564	0, 2564	0, 1022	24, 1185	0, 1185
Geometric Mean (GSD)	299.7 (5.18)	491.9 (3.24)	340.5 (4.65)	168.2 (4.54)	267.3 (4.06)	184.5 (4.41)
95% CI of Geometric Mean[1]	154.3, 582.4	199.3, 1213.8	200.8, 577.2	93.5, 302.5	73.2, 976.4	110.9, 307.2
P-Value [2]	0.0004	0.0034	< 0.0001	0.0003	0.0404	< 0.0001
Responders (%) [3]	13 (50.0%)	3 (33.3%)	16 (45.7%)	7 (25.0%)	2 (28.6%)	9 (25.7%)
95% CI for Responders (%)[4]	29.9 %, 70.1%	7.5%, 70.1%	28.8%, 63.4%	10.7%, 44.9%	3.7%, 71.0%	12.5%, 43.3%

Note: N = Number of subjects in the prospective cohort. ; GSD=Geometric Standard Deviation; CI=Confidence Interval.

[1] CI is estimated using a two-sided Normal Approximation Method.

[2] Based on a regression analysis with baseline in the model using a 0.05% significance level testing that the slope equals one.

[3] A responder was defined as a subject whose value was greater than the baseline value and greater than the following threshold values: HPV-16: 455; HPV-18: 450.

The denominator for percentages is based on the total number of subjects (n) with a result at the visit being summarized.

[4] CI is estimated using a two-sided Exact (Clopper-Pearson) Method.

Table S7: Number of HPV + Memory B Cells per Million of Total IgG Secreting B cells in Retrospective Cohorts.

	Memory B Cells (Number of HPV specific memory B cells/per million total IgG secreting cells)		
	**Retrospective Cohort with 2 prior HPV Vaccinations (N=74)**	**Retrospective Cohort with 3 prior HPV Vaccinations (N=72)**	**Difference 3 Prior - 2 Prior**
**HPV-16**			
n	74	72	N/A
Min, Max	0, 2444	0, 2701	N/A
Geometric Mean (GSD)	301 (4)	241 (4)	0.8 (4) [6]
95% CI of Geometric Mean [1]	218, 428	169, 343	0.49, 1.28 [6]

**P-Value [2]**			**0.3369**
Seropositive (%) [3]	38 (51.4%)	24 (33.3%)	-18.0% [7]
95% CI for Seropositive (%) [4]	39.4%, 63.15%	22.7%, 45.43%	-33.8%, -2.3%[7]
**P-Value [5]**			**0.0277**
**HPV-18**			
N	74	72	N/A
Min, Max	0, 1726	0, 610	N/A
Geometric Mean (GSD)	90 (5)	74 (4)	0.8 (5) [6]
95% CI of Geometric Mean [1]	62, 129	53, 104	0.5, 1.4[6]
**P-Value [2]**			**0.4545**
Seropositive (%) [3]	10 (13.5%)	1 (1.4%)	-12.1% [7]
95% CI for Seropositive (%) [4]	6.7%, 23.5%	0.0%, 7.5 %	-20.4%, -3.9%[7]
**P-Value [5]**			**0.0055**

Note: Only subjects who were responders are summarized GSD=Geometric Standard Deviation; CI=Confidence Interval. N/A=Not applicable.

[1] Confidence Interval (CI) is estimated using a two-sided Normal Approximation Method.

[2] P-value is based on a two-sample t-test using a 2-sided 0.05% significance level.

[3] A responder was defined as a subject whose value was greater than the following threshold values: HPV-16: 455; HPV-18: 450. The denominator for percentages is based on the total number of subjects (n) with a result at the visit being summarized.

[4] CI is estimated using a two-sided Exact (Clopper-Pearson) Method.

[5] P-value is based on a two-sample Chi-square test using a 2-sided 0.05% significance level.

[6] Difference is the relative difference. A value of one means the response after two doses is the same as the response after three doses.

[7] Difference is the absolute difference
